# Evaluation of contact heat thermal threshold testing for standardized assessment of cutaneous nociception in horses - comparison of different locations and environmental conditions

**DOI:** 10.1186/1746-6148-9-4

**Published:** 2013-01-08

**Authors:** Christin Poller, Klaus Hopster, Karl Rohn, Sabine BR Kästner

**Affiliations:** 1 Clinic for Horses, University of Veterinary Medicine Hannover, Foundation Hannover, Germany; 2 Department of Biometry, Epidemiology and Information Processing, University of Veterinary Medicine Hannover, Foundation Hannover, Germany; 3 Clinic for Small Animals, University of Veterinary Medicine Hannover, Foundation Hannover, Germany

**Keywords:** Horse, Nociception, Pain, Contact heat, Environmental condition

## Abstract

**Background:**

The aim of the study was to evaluate the performance of contact heat thermal stimulation in horses at different body sites and under different environmental conditions and different test situations. Five warm-blood horses were equipped with the thermal probe located on the skin of nostril (N), withers (W) or coronary band (C). Skin temperature and reaction temperature (thermal threshold) at each location were measured and percent thermal excursion (% TE = 100 * (threshold temperature - skin temperature)/(cut-out temperature - skin temperature) was calculated. Environmental conditions were changed in partial random order for all locations, so each horse was tested in its familiar box stall and stocks, in the morning and evening and at warm and cold ambient temperatures. Type of reaction to the stimulus and horse’s general behaviour during stimulation were recorded. The stimulation sites were examined for the occurrence of possible skin lesions.

**Results:**

Skin temperatures were significantly different during warm and cold ambient temperatures at all three locations, but remained constant over repeated stimulation. An obvious response to stimulation before reaching cut-out temperature could be detected most frequently at N and W in boxes during warm ambient temperatures. The most frequent type of reaction to thermal stimulation at the nostril was headshaking (64.6%), skin twitching at the withers (82.9%) and hoof withdrawal at the coronary band (79.2%).

**Conclusion:**

The outcome of thermal threshold testing depended on ambient temperature, stimulation site and environment. Best results with the WTT2 in horses were obtained at the nostrils or withers in a familiar environment at warm ambient temperatures.

## Background

Repeatable and reliable tests for detection and quantification of analgesia are essential for development of effective analgesic protocols. Nociceptive threshold tests are standard models of nociceptive pain in laboratory animals [1]. Among different stimulation modalities heat stimuli are widely used in classic tests [2-4]. In horses, contact heat and radiant heat have been used as a quantifiable stimulus to determine temperature thresholds [5-8] or the latency between stimulation and response [9-12]. A thermode based system for determination of thermal nociceptive thresholds, initially designed and validated for use in cats [13], has been adapted for the use in horses [5-8]. When using such systems, definition of a clear cut end-point of stimulation, such as skin twitching, shaking or hoof withdrawal is crucial for reliable and repeatable determination of the nociceptive threshold [14]. Such behavioural end-points can be reflex related or may represent conscious perception of pain dependent on the stimulated body region, thereby, influencing the level of the response threshold. When working with large animals outside a controlled laboratory environment, detection of an expected end-point can be difficult because of a wide range of environmental conditions. Seasonal and daily fluctuations in temperature may influence nociceptive threshold testing via both animal factors (skin temperature, perfusion and moisture content) and interference with the equipment itself [14-17]. The results of nociceptive threshold testing could be influenced by housing the horses close to each other [18]. Often algesimetric studies are performed by separating individual animals in stocks [5,6,14] or by restraining them by use of a halter [11,19]. This may introduce confounding variables, such as the stress of moving them away from their familiar surroundings to a different environment and disruption of social bonds or the restraint itself may lead to abnormal behaviour and altered responses [18]. A further difficulty can be the potential boredom during prolonged testing schedules when horses become easily distracted while standing in stocks [17].

Therefore, the aim of the study was to compare thermal nociceptive thresholds and the performance of a thermode based contact heat test system at different body regions under different environmental conditions and different day times in restrained or free moving horses.

## Results

### Acceptance of the testing device

All five horses tolerated attachment of the measurement equipment without any problems. One horse reacted with head shaking and skin twitching when the air bladder at the nostril was inflated, however, two minutes after inflating the bladder, thermal stimulation was possible without any distracting factors. When the testing device was disconnected from the belt, one of the five horses was frightened by the noise from the Velcro strip.

### Skin temperature

Before each measurement the thermal probes were moved to a new area of skin.

**Figure 1:**
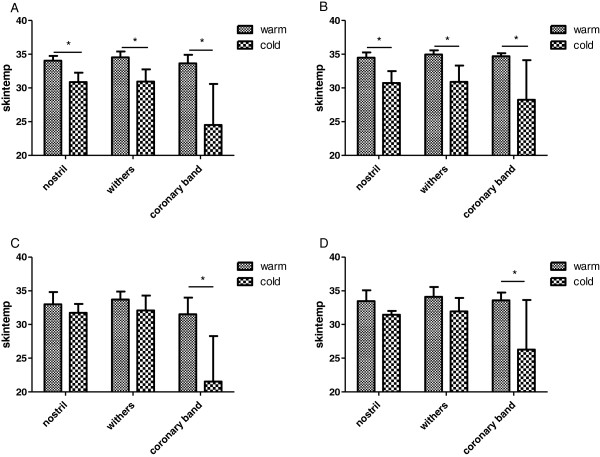
**Influence of ambient temperature and different stimulation conditions on skin temperature.** Skin temperatures were measured before each thermal stimulation. All diagrams show the differences in skin temperature during warm (> 20°C) and cold (< 10°C) ambient temperatures at three different body parts of the horse (nostril, withers, coronary band) under different stimulation conditions: **A:** free moving horse in a box stall in the morning. **B:** free moving horse in a box stall in the evening. **C:** horse restrained in stocks, in the morning. **D:** horse restrained in stocks, in the evening.

Skin temperature did not change over the three consecutive measurements at 20 minute intervals. The ambient temperature and test location influenced the skin temperature. At warm ambient temperature (>20°C) there was no significant difference between the three locations nostril, withers and coronary band. When ambient temperature was less than 10°C, skin temperature at the nostril and withers was significantly higher than at the coronary band. Overall skin temperature was significantly lower during cold ambient temperatures at all three locations (Figure 1A, B). When the horses were restrained in stocks, skin temperature was significantly lower in cold ambient temperature than at warm temperatures at the coronary band only (Figure 1C, D). Different day times (morning/evening) had no influence on skin temperature.

### Thermal threshold

**Figure 2:**
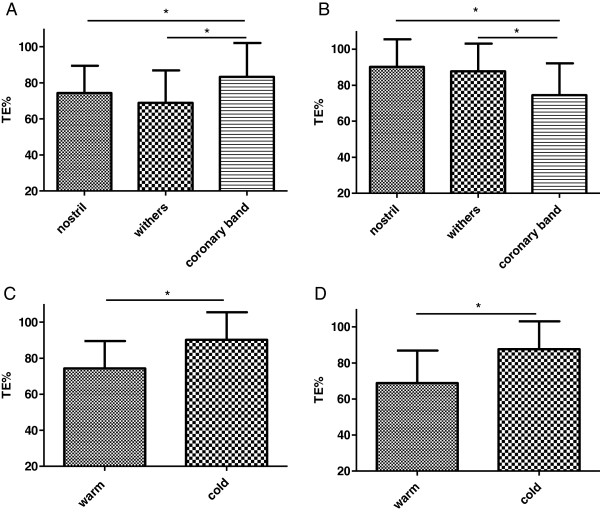
**Influence of ambient temperature and different stimulation conditions on percentage thermal excursion.** All diagrams show percentage thermal excursion (TE % = 100 × ([T_T_ – T_0_]/[T_c_-T_0_]), T_T_ is the thermal threshold temperature, T_0_ is the skin temperature and T_c_ is the thermal cut-out temperature (Brosnan et al. 2009)). These results were formed without differentiating of place or day time. **A**: TE % at three different body parts (nostril, withers, coronary band). Thermal stimulation was performed during warm ambient temperature. **B:** TE % at three different body parts (nostril, withers, coronary band). Thermal stimulation was performed during cold ambient temperature. Data of 13 measurements at the coronary band had to be excluded because neither end-point nor cut-out temperature could be reached. **C:** TE % was compared between warm and cold ambient temperatures during measurements at the nostril. **D:** TE % was compared between warm and cold ambient temperatures during measurements at the withers.

Threshold temperature and TE % were stable over the 3 consecutive measurements and were mainly influenced by ambient temperature and localisation of the probe (Figure 2 A-D). The horses responded at lower temperatures to the thermal stimulus at the nostril (*p* = 0.0074) and withers (*p* = 0.0025) during warm ambient temperatures compared to cold ambient temperatures (Figure 2A-D). In contrast, at the coronary band TT and TE % were significantly higher at warm ambient temperatures compared to cold ambient temperatures (Table 1).

**Table 1:** Comparison of threshold temperature

		**Warm ambient**	**Cold ambient**	**p-value**
		**Temperature (n = 60)**	**Temperature (n = 47)**	
Nostril	TT (°C)	51.1 ± 3.40	54.5 ± 3.90	0.0376
	TE %	74.3 ± 15.2	90.2 ± 15.3	0.0074
Withers	TT (°C)	49.9 ± 4.00	53.9 ± 3.80	0.0164
	TE %	68.9 ± 18.0	87.7 ± 15.4	0.0025
Coronary band	TT (°C)	53.2 ± 4.30	49.3 ± 6.30	0.0004
	TE %	83.3 ± 18.8	74.5 ± 17.7	0.0097

Mean ± standard deviation of threshold temperature (°C) and percentage thermal excursion (TE %) at the nostrils, withers and coronary band of 5 horses during warm and cold ambient temperatures (n = 60, without differentiation of place or time of the day). Data of 13 measurements at the coronary band during cold ambient temperatures (n = 47) had to be excluded because neither end-point nor cut-out temperature could be reached.

### End point reaction/behaviour during measurements

The most frequent reaction to thermal stimulation at the nostril was head shaking (64.6%) followed by rubbing the nose (35.4%). Stimulation at the withers produced skin twitching (82.9%) and less commonly a whole body shake (9.8%) or turning the head (7.3%). All horses reacted to the thermal stimulus at the coronary band with a leg lift (stamping) (79.2%) or rubbing (20.8%) (see Additional file 1).

In between stimulations the horses showed typical behaviour in their box stalls such as eating hay and snoozing. In distracted or anxious horses (head elevated with ears in forward position), the cut-out temperature was often reached without a response, leading to a “failed” stimulation.

### End point detection

Clear end point detection was most successful when tests were performed in a box stall during warm ambient temperature at the nostril and the withers (Table 2).

**Table 2:** Occurrence of end point detection during thermal stimulation

**Place**	**Temperature**	**Success rate [%]**	**Nostril**	**Withers**	**Coronary band**
			**(n = 30)**	**(n = 30)**	**(n = 30)**
Box	Warm ambient temperatures	Failure	6.66^a^	3.34^a^	40.0^b^
		Success	93.4^a^	96.6^a^	60.0^b^
	Cold ambient temperatures	Failure	50.0^a^	43.4^a^	33.4^a^
		Success	50.0^a^	56.6^a^	66.6^a^
Stocks	Warm ambient temperatures	Failure	13.4^a^	26.6^abA^	50.0^bB^
		Success	86.6^a^	73.4^abA^	50.0^bB^
	Cold ambient temperatures	Failure	70.0^a^	56.6^ab^	43.4^b^
		Success	30.0^a^	43.4^ab^	56.6^b^

Success rate (%) of end-point detection in response to thermal stimulation in 5 horses housed in a box stall or standing in stocks. Thermal stimulus was given to three different body parts (nostril, withers, coronary band) and during two different ambient temperatures (< 20°C or < 10°C). The overall number of stimulations was n = 30.

‘success’ – clear, visible reaction to the thermal stimulus before reaching cut out (threshold < cut-out temperature (56°C)). ‘failure’ - no visible reaction to the thermal stimulus and cut-out (56°C) was reached.

Comparison between locations: nostril/withers, nostril/coronary band, withers/coronary band.

^a, b, c^ = values with unequal superscript numbers were significantly different (*p* < 0.05).

^A, B, C^ = values with different superscript numbers had a statistical trend (*p* < 0.1).

### Skin lesions

After completion of all measurements in 7 of 40 (17.5%) and in 2 of 40 (5%) thermal stimulations the skin area at the stimulation site was mildly raised without being painful on palpation, at the nostril and the withers, respectively. The day after the experiment the stratum corneum excoriated at the nostrils, which only occurred after thermal stimulation at warm ambient temperatures and when cut-out was reached. The typical skin pigmentation reoccurred 3 – 5 days after the experiment. There were no skin alterations at the coronary band.

## Discussion

Contact heat thermal stimulation is suitable for standardized assessment of thermal cutaneous nociception in horses. However, the success of clear end point detection can be variable and depends on the body region stimulated, ambient temperatures and the test conditions.

Ambient temperature appeared to have significant influence on thermal threshold testing. In the present study thermal threshold temperatures at the nostril and withers were significantly lower at warm ambient temperature compared with cold temperatures. There is little information in the literature concerning the influence of ambient temperatures or skin temperatures and on determined thermal thresholds in horses. Tail flick latencies (TFL) in rats and mice were significantly increased and tail skin temperature was decreased when the tail was immersed in cold water (0°C, 5°C or 10°C) before testing [15,20]. The current study indicated that skin temperature was strongly correlated with ambient temperature. Ambient temperature below 10°C resulted in significantly lower skin temperature but higher reaction temperatures compared to ambient temperatures above 20°C. It might be necessary to heat the probe up to a higher temperature to get heat transferred and reach the nociceptive threshold when the skin tissue is at a lower temperature. There was no increase in skin temperature over the three consecutive measurements, showing that the 20 minute interval was adequate for the skin to return to its normal temperature without active cooling. Cold ambient temperature and therefore low skin temperatures (13.9 to 21.9°C) resulted in prolonged heating time and reduced power of the thermal threshold testing device. So cut-out temperature couldn’t be reached in several measurements at the coronary band and the heating cycle was interrupted at 48 – 50°C. The data of the inaccurate measurements were excluded from analysis and therefore 47 thermal thresholds instead of 60 TT were considered. For further studies being performed at cold ambient temperatures more powerful probes and batteries need to be used.

The randomized delay in starting thermal stimulation in the current study allowed the duration of one heating cycle to be randomly varied. This prevented the horses from becoming conditioned to any audible clue such as a click when heating was started. It also ensured the assessor did not become accustomed to the normal time lapse from start of stimulation to response. Some studies suggest that horses can get conditioned to the noxious stimulus. They reacted as soon as they saw the lamp supplying radiant heat [10] or as they felt the stimulus before it became painful [17]. In the present study thermal thresholds did not change when thermal stimulation was repeated at 20 minute intervals, suggesting that horses did not become accustomed to the noxious stimulus. In a further study which is in preparation for publication (Poller C, Hopster K, Kästner SBR) TT did not change over 10 repeated measurements in 30 minute to 2 hours intervals when horses were treated with a placebo (saline solution). Similar results were reported in another study where thermal thresholds remained constant over 24 hours [19].

The quick heat transfer to nociceptors is an important factor contributing to successful clear end-point detection and preferably low thresholds to prevent skin burns. Heavily pigmented epidermal tissues and hair covering impeded the relative transparency to near infrared light [21] and probably the thermal transfer in deeper layers. It was possible that hairy skin, despite clipping, isolated the deeper layers of epidermis from the heat source [14] leading to the conclusion that this could have contributed to the late or failed response to the thermal stimulus at the coronary band. Slower heating rates could improve the heat conduction to the nociceptors by warming up the skin for longer excursion but also increased the likelihood of skin burning. Unfortunately, it was not possible to perform the current study in a fully randomised design concerning experiments at warm and cold ambient temperatures for seasonal and logistic reasons as climatized rooms were not available. Therefore, possible conditioning of the horses to the thermal stimulus in the second set of tests (cold temperatures) could be considered. In case of conditioning, lower thermal thresholds at cold ambient temperatures compared to warm ambient temperatures would have been expected, but results showed the opposite with higher TT in November.

All horses were trained wearing the equipment to ensure that their reaction to thermal stimulation was not influenced by discomfort or stress. The horses developed individual behaviour when they were restrained in stocks: some of them became bored and distracted whilst others were very nervous or anxious. Nociceptive thermal thresholds were not constant or reliable when thermal stimulation was applied to horses tied in stocks, resulting in high thresholds reaching cut-out temperature. Different authors suggested that nociceptive threshold testing in horses should be performed within the animal’s normal environment and when unrestrained to avoid distraction of the horse [14]. To the author’s knowledge so far there is only one more published study with thermal threshold testing in unrestrained horses [22].

When visible end-points at the different body sites (nostril, withers, coronary band) were compared, the reaction to the stimulus at the withers was the most clear and easy to identify, as the transformation of the noxious stimulus into a visible reaction is mainly mediated via a reflex pathway (skin flick response via the spinothalamic tract) [23]. Coordinated head shaking or rubbing the nose against an object requires conscious perception (trigeminal nerve at the nostrils) with more individual variation [23]. In this study, measurements at the coronary band had the lowest reliability in producing constant reactions to thermal stimulation. It has been suggested that the depth and density of Aδ and C-fibres and the distribution of nociceptors may be variable between species and body parts [14]. The epidermis in horses has been reported to be nearly twice as thick as in cats or rodents [24]. In addition to the interspecies differences in epidermal thickness there were also variable data between body parts within species [24]. It is likely that nociceptors and nerve fibres in horses are located in deeper layers. This would lead to higher threshold temperatures recorded at the skin surface, as seen in the present study (49.9 ± 4.0°C at the withers), compared to cats or rodents (approximately 45°C) [13,25] assuming that temperature equilibration between skin surface and nociceptors needed longer time in thick skin. Thermal threshold might also be affected by blood flow [14]. Measurements of blood flow demonstrated significant differences between species and body parts [25]. Failed reactions to a thermal stimulus on the distal limb in sheep at ambient temperatures below 8°C were considered as result of vasoconstriction in the skin and ischaemia of the small nerve fibres [17]. It is likely that thermal stimulation at the coronary band of horses was probably affected by skin thickness, increased hair density and reduced blood flow, and, particularly at low ambient temperature, by vasoconstriction. A more proximal part of the limb as stimulation site, like mid cannon bone instead of the coronary band, might be less affected by temperature changes and blood flow allowing more successful end-point detection.

The uneven gender distribution and the lack of knowledge of the state of the estrous cycle in the 3 mares is a limitation of the study and might have influenced the results, because in other species, including humans, gender can influence nociceptive sensitivity. Women were more sensitive to cold, heat and ischaemic pain than men [26] or in female rats nociceptive sensitivity was decreased after ovariectomy [27]. However, the influence of the estrous cycle on nociception and pain was controversial in these studies [26,27]. To the author’s knowledge there are no published studies in horses comparing thermal nociceptive thresholds between mares and geldings or the influence of the estrous cycle. As the season might influence the occurrence of the oestrous in mares, it cannot be ruled out that the differences in thermal thresholds between warm and cold temperatures were also influenced by hormonal changes and are not only associated with temperature differences.

## Conclusion

This study demonstrated that many factors influenced the thermal threshold measured in horses, with body part and environmental conditions. Conditions resulting in the most successful measurements were environmental temperatures of 20°C or higher, thermal stimulation at the nostril or withers and testing with the horse unrestrained in a box stall. The results illustrate that it is important to state under which conditions thermal stimulation experiments are executed and that conditions should be uniform throughout the entire study.

## Methods

### Animals

The study was approved by the Ethics Committee for Animal Experiments of Lower Saxony (33.12-42502-04-10/0136). Five warm-blood horses (2 geldings and 3 mares) weighing 550 +/− 50 kg, ranging from 5 to 17 years were used in the present study. All of them were determined to be healthy on the basis of results of a clinical examination and were free of chronic lameness. During the measurement horses were housed in their home box stall (4 × 4 m) without open access to pasture. They had free access to hay and fresh water.

### Experimental design

The study was performed in a complete crossover design (Latin square). Stimulation conditions were randomized, except ambient temperature. Each horse went through 24 different stimulation conditions including 3 different body sites (nostril, withers, coronary band), 2 different ambient temperatures (> 20°C; < 10°C), 2 different day times (morning (6 a.m. to 11 a.m.), evening (3 p.m. to 9 p.m.)) and free moving in a familiar box stall or standing in stocks. Three repeated measurements were performed under each stimulation condition. Experiments with warm ambient temperatures (> 20°C) were performed in July 2010 and with cold ambient temperatures (< 10°C) in November 2010.

### Instrumentation

**Figure 3:**
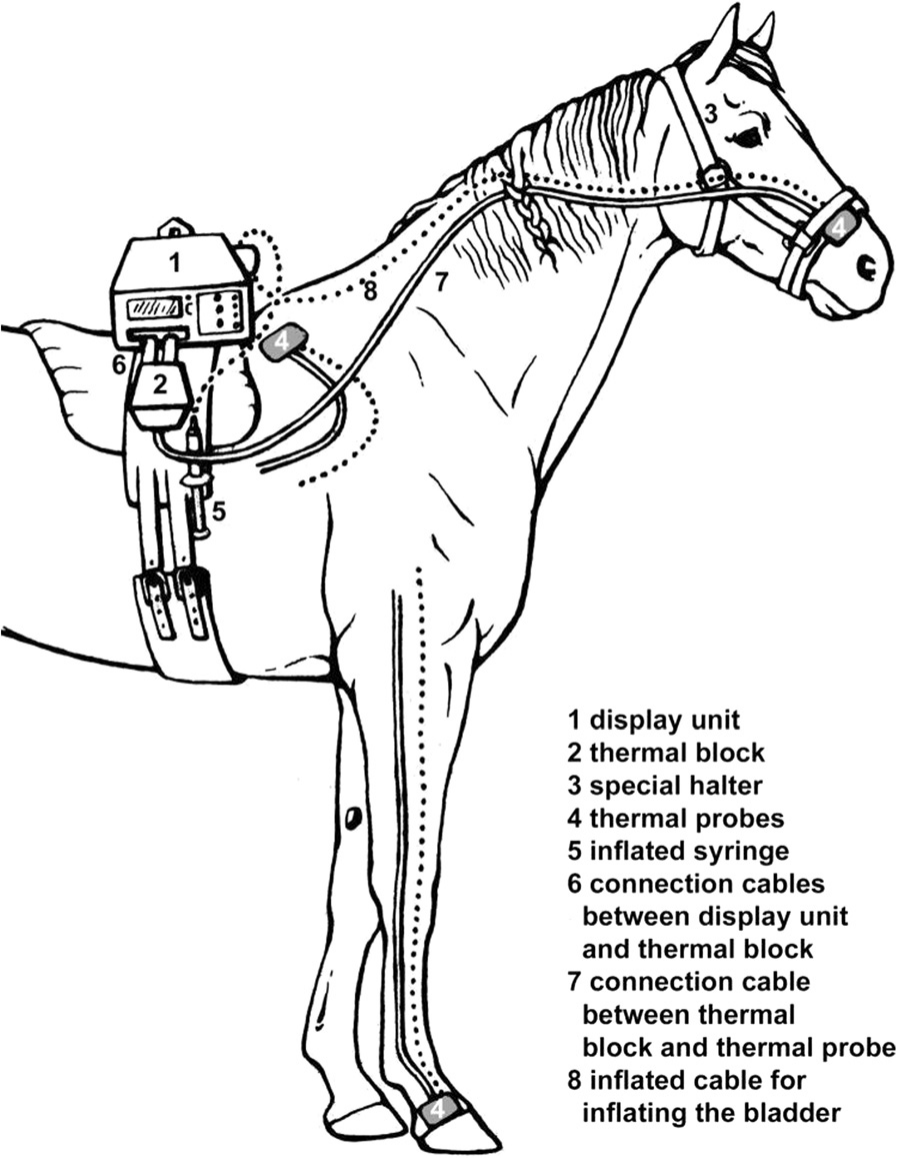
**Attachment of the Wireless Thermal Threshold testing system to the horse.** The thermal probes (4) were placed at three different body parts of the horse (nostril, withers, coronary band).

The horses were equipped with a previously validated wireless thermal threshold testing system (Wireless Thermal Threshold Testing System (WTT2), Topcat Metrology Ltd., Ely UK) [7]. The display unit and heating block were mounted on the withers of the horse with the help of a surcingle and Velcro strips. The thermal probe containing the heating and temperature sensing element was placed at the skin above the nostrils or an area of shaved skin at the withers or coronary band of a front leg (Figure 3). The skin was shaved with a razor blade the day before the experiment to avoid acute skin irritation. The contact pressure of the thermode was held constant at approximately 80 mmHg by an inflatable bladder between the probe and Velcro® strips and duct tape (nostril, withers) or duct tape and an elastic bandage (coronary band). For measurements at the nostril a special halter with a Velcro strip was used to fix the probe in position. At the withers and the coronary band the heating probe was fixed by adhesive tape and bandages. During the measurement at the coronary band the ribbon and the inflating tube were fixed to the leg by an elastic Velcro strip. Especially while thermal stimulation was performed the examiner looked for distracting insects and when necessary horses were sprayed with an insect repellent (Power Phaser, leovet).

### Stimulation protocol

All horses were familiar with the testing device. The thermal probe was placed on the stimulation site at least 5 minutes before testing to allow temperature equilibration between the skin and the probe. The heating rate was set at 0.6°C/s for stimulation at the withers and the coronary band and 0.8°C/s for stimulation at the nostrils because the lower rate produced significant burns in a pretrial. A maximum probe temperature (cut-out) of 56°C was chosen for all tests. The skin temperature was recorded and the probe activated via infrared remote control. The heat controller was set to start with a random delay, so the time before heating varied and therefore neither horse nor operator knew when heating began. Heating was stopped and the temperature recorded when the horse shook its head or rubbed the nose against an object or its legs, a skin twitch (reflex contraction of the cutaneous trunci muscle) occurred or the horse turned its head, and a leg lift (hoof withdrawal reflex, HWR), stamping or pawing was observed with stimulation at the nostrils, the withers and the coronary band, respectively. The type of reaction to the thermal stimulus was documented. When there was no clearly discernable reaction to the stimulus the measurement was assessed as ‘failure’ otherwise it was a ‘success’. The position of the head, the ears and the nostril were recorded to assess whether the horse was nervous or distracted. If there was no reaction, the cut-out temperature was recorded. After each heating process, the probe was removed from the skin to allow cooling, and the probe was moved to a new area of skin for the next measurement. Stimulation was repeated 3 times at each location under each condition with 20 minute intervals between each stimulation. After each test session, the skin was examined for swelling and skin lesions.

### Statistical analysis

Normal distribution of the data was approved by visual assessment of the q-q-plots of the model residuals. Data are reported as mean ± standard deviation.

Thermal thresholds were analysed as reaction temperature (thermal threshold TT), and in addition as percentage of thermal excursion (TE %):

$$\text{TE}\%=100\times \left(\left[T_{T}–T_{0}\right]/\left[T_{c}-T_{0}\right]\right)\text{.}$$

Where T_T_ is the thermal threshold temperature, T_0_ is the skin temperature and T_c_ is the thermal cut-out temperature [28].

Influence of ambient temperature, place of examination, body site of the horse and time of day were analysed using a four way analysis of variance (ANOVA) with repeated measurements within subjects and post-hoc Tukey-Kramer tests for multiple (pair wise) comparisons. Data from environmental conditions without significant influence on thermal threshold temperatures were pooled and reanalysed by analysis of variance. The Fisher’s exact test was used to analyse categorical results between counts of stimulations with and without (reaching cut-out) a clear end point defined as the’success rate’ of thermal stimulation. Statistical significance was attributed when *p* < 0.05. Analyses were carried out with the statistical software SAS®, version 9.2 (SAS Institute, NC, USA).

## Abbreviations

WTT2: Wireless Thermal Threshold testing device 2 (modified for horses); N: Nostril; W: Withers; C: Coronary band; TT (°C): Thermal thresholds in°C; TE %: Percentage of thermal excursion; HWR: Hoof withdrawal reflex; TFL: Tail flick latency.

## Competing interests

The authors declare that they have no competing interests.

## Authors’ contributions

SBRK conceived the project. CP accomplished the practical part of the study and was supported from KH and SBRK. KR carried out the statistical analysis. SBRK, KH and CP participated in interpretation of the study results. CP drafted the paper which was later revised by all co-authors through substantial contributions to the content of the paper. All authors read and approved the final manuscript.

## Supplementary Material

Additional file 1 **Reaction to thermal stimulation.** This video shows typical reactions to noxious thermal stimulation at the nostril, withers and coronary band. Click here for file
